# Secure attachment may foster psychological momentum through building enduring happiness: insights from an exploratory cross-sectional study involving regular exercisers

**DOI:** 10.3389/fpsyg.2025.1671289

**Published:** 2025-10-29

**Authors:** Walid Briki, Keith D. Markman

**Affiliations:** ^1^Centre Hospitalier de Grasse, Grasse, France; ^2^Ohio University, Athens, OH, United States

**Keywords:** attachment theory, momentum, psychological well-being, goal pursuit, physical exercise

## Abstract

Past research has shown that secure attachment promotes hedonic or fluctuating forms of happiness by enhancing perceptions of goal progress. The present study extends this work by examining authentic-durable happiness—a more stable, peace-oriented form of well-being—and by positioning psychological momentum (PM) as an experiential construct of exploration. Regular exercisers (*N* = 511) completed online questionnaires assessing secure attachment, authentic-durable happiness, and PM over the previous four weeks. To clarify the interrelationships among these constructs, we tested two competing structural equation models. Model 1, grounded in a hedonic perspective, specified PM as a mediator between secure attachment and authentic-durable happiness. Model 2, informed by eudaimonic and self-determination theory perspectives, specified authentic-durable happiness as a mediator between secure attachment and PM. Results indicated stronger support for Model 2: Authentic-durable happiness partially mediated the relationship between secure attachment and PM. These findings highlight authentic-durable happiness as a key psychological resource linking secure attachment to sustained engagement. By contrasting hedonic and eudaimonic perspectives, the study advances attachment theory beyond fluctuating happiness and provides preliminary evidence that secure attachment may foster enduring well-being, which in turn may energize perceptions of PM. Overall, this work introduces PM as a phenomenological manifestation of exploration and underscores the value of examining both transient and durable happiness in understanding the motivational impact of secure attachment.

## Introduction

Attachment refers to the propensity to build strong emotional connections with specific people who play the role of nurturing caregivers, called “attachment figures” (e.g., parents, romantic partners) (Bowlby, 1978, 1982; Melges and Bowlby, 1969). *Secure attachment*, viewed as the consequence of satisfying the basic need for safety, refers to the feeling that one is safe, that one can rely on close others when needed, and that one is worthy of unconditional love (Bowlby, 1978, 1982). It arises when caregivers show interest in the individual and validate and support the pursuit of desired goals (Jakubiak and Feeney, 2016, 2017). Under such conditions, people experience both emotional comfort and confidence in their abilities to deal with the external world’s demands, thereby giving rise to the desire to engage with the surrounding environment (Bowlby, 1978, 1982).

## Attachment theory, psychological momentum, and happiness

### Goal progress: a way to capture exploration

According to attachment theory, *exploration* refers to “…a basic drive to engage autonomously with the environment and to pursue goals throughout the life span” (Jakubiak and Feeney, 2016, p. 317). This drive is thought to be associated with a wide range of positive consequences such as happiness and well-being (Bowlby, 1978, 1982; Jakubiak and Feeney, 2016, 2017), consistent with the notion that making progress toward desired goals should promote positive affect (Carver and Scheier, 2000; Rasmussen et al., 2006; Wrosch et al., 2003). Jakubiak and Feeney (2016) suggest that successful and autonomous engagement with the physical or social environment can be represented by the notion of *goal progress*.

Empirically examining relationships between spousal attachment, perceived goal progress, and well-being, Jakubiak and Feeney (2016) found that: (a) daily secure attachment (induced by support from the spousal partner) and perceptions of daily goal progress positively predicted *hedonic well-being* (also called “subjective well-being”)—assessed through the measurement of positive and negative emotions; (b) daily secure attachment positively predicted perceptions of daily goal progress; and (c) perceptions of daily goal progress partially accounted for the relationship between daily secure attachment and hedonic well-being. A recent meta-analysis confirmed the link between adult attachment and hedonic well-being across diverse populations (Zhang et al., 2022). Briki (2018) also examined the association between perceived goal progress and hedonic well-being in a physical exercise setting, and found that higher levels of feeling that one is making progress toward exercise goals positively predicted hedonic well-being. Taken together, these findings support the view that people successfully engage autonomously with their environments—and, thus, make progress toward their goals—and feel happier whenever they feel cared for and supported by close others (e.g., Bowlby, 1978, 1982).

In the present study, we posit that *psychological momentum* (PM) might be an important aspect of the phenomenology of exploration, as it represents not only goal progress but also an adaptive integration of affect, cognition, and behavior (Briki and Markman, 2018; Honey et al., 2023). Indeed, whereas goal progress refers primarily to discrete perceptions of moving forward, PM captures a richer experiential state. It integrates affective, cognitive, and motivational components into a dynamic sense of “being carried forward,” making it qualitatively distinct from simple progress appraisals. This distinction matters for attachment theory, because the sense of safety afforded by secure attachment may support not only incremental progress but also the broader, self-sustaining subjective experience of engagement that PM reflects.

### Psychological momentum is more integrative than goal progress

PM is best understood as a multidimensional phenomenon rather than a unidimensional construct of achievement (Gernigon et al., 2010; Taylor and Demick, 1994; Vallerand et al., 1988). It reflects the subjective experience that progress is not only occurring but also accumulating in a way that energizes and sustains further action (Briki and Markman, 2018; Iso-Ahola and Dotson, 2016; Honey et al., 2023).

In the context of physical exercise, PM can refer to situations where the exerciser feels that things are going well regarding their fitness goals, such as feeling satisfied with noticeable improvements (e.g., “*I’m thrilled that my efforts are paying off! I’ve lost weight in just four weeks!*”). PM is understood as the subjective experience of goal pursuit (Briki and Markman, 2018) and has been defined as “… a psychological force in which several factors or qualities converge in a synergistic way to enable one to perform at a level not ordinarily possible” (Iso-Ahola and Dotson, 2014, p. 20). Iso-Ahola and Dotson (2016) proposed that an initial success might activate the PM process, which involves three key mental stages: (a) a boost in self-confidence, where individuals attribute success to their abilities and efforts (i.e., psychological energization); (b) heightened expectations of future success (i.e., psychological impetus); and (c) a shift in mental state, characterized by a feeling of unstoppable progress (i.e., subjective experience of PM). Once activated, the PM process is believed to enhance both mental focus and physical effort, driving individuals to continue pursuing their goals with renewed vigor (Iso-Ahola and Dotson, 2016; Briki and Markman, 2018).

Although goal progress can indicate achievement—and to some extent, exploration—PM offers a more comprehensive and impactful framework for capturing engagement within achievement contexts. Whereas goal progress primarily reflects perceived movement toward a specific target, PM captures a dynamic, holistic experience that involves a wide range of emotions, cognitions, and behaviors (Gernigon et al., 2010; Honey et al., 2023; Taylor and Demick, 1994; Vallerand et al., 1988). This broader perspective positions PM as a more embodied and meaningful construct of human engagement. Moreover, PM accounts for the phenomenological depth of individual experience in ecological achievement settings (Briki and Markman, 2018)—an aspect that goal progress alone fails to reflect. Importantly, PM also includes three key experiential characteristics—frequency, intensity, and duration—that are known to shape both ongoing actions and subjective experiences (Iso-Ahola and Dotson, 2014, 2016). These features of PM offer a more complex and nuanced understanding of the overall engagement experience than does the concept of goal progress alone. Thus, to better capture engagement in achievement contexts, we argue that PM represents a more suitable and theoretically sound focus when examining the lived experience of exploration. In this study, we posit that secure attachment may influence the experiential dimensions of PM by encouraging repeated engagement (frequency), enhancing the salience of progress (intensity), and sustaining motivation over time (duration).

### Psychological momentum and happiness

Because PM constitutes a self-regulation mechanism that is thought to facilitate task completion (Briki and Markman, 2018; Iso-Ahola and Dotson, 2016), it can be viewed as a phenomenon driven by the satisfaction of psychological needs and linked to psychological well-being. This study aims to examine PM from the perspective of attachment theory and to explore its potential relationship with happiness. Recently, the study of happiness—more specifically, of its processes and conditions of development—has gained prominence (e.g., Diener et al., 2015; Diener and Tay, 2015; Li et al., 2021; Myers and Diener, 2018; Oishi et al., 2012). A survey of the literature reveals that the concept of happiness might be differently conceptualized depending upon self-construal dimensions (Dambrun, 2017; Dambrun et al., 2012; Monacis et al., 2021). Dambrun and colleagues posit that the conception of self as an interdependent entity, reflecting the perception of being highly connected with others and the physical environment, precipitates the development of selfless functioning—characterized by self-transcendence—that gives rise to *authentic-durable happiness* (Dambrun et al., 2012; Monacis et al., 2021). In this case, people are motivated to satisfy psychological needs, to self-actualize, and to function in an optimal or harmonious way within their social and physical environments, thereby leading them to experience greater mindfulness and more durable states of contentment (e.g., pleasure, joy) and inner peace (e.g., peace of mind, serenity).

Most prior work linking attachment and well-being has focused on hedonic or fluctuating happiness (e.g., Jakubiak and Feeney, 2016). Our study extends this literature by focusing on authentic-durable happiness, which emphasizes inner peace and enduring contentment rather than transient affective shifts. Our interest in that form of happiness was inspired by the fact that it is a robust predictor of mental health variables such as meaning in life, sense of coherence, optimism, perceived resiliency, and low rumination (Dambrun et al., 2012; Monacis et al., 2021). By contrasting these two conceptions of happiness, we aim to provide a more critical account of how secure attachment may foster not only immediate positive affect but also deeper and more sustainable forms of well-being. In the present study, we expected secure attachment to promote authentic-durable happiness.

## Two plausible hypotheses

The primary goal of the present study was to examine PM from the scope of attachment theory, and to test two competing models linking secure attachment, authentic-durable happiness, and PM. These models build on two contemporary but distinct perspectives in the literature of well-being.

### Hypothesis 1 (model 1: the hedonic perspective)

Research on hedonic or subjective well-being suggests that feeling secure in close relationships facilitates perceptions of progress toward valued goals, which in turn generates positive affect. Jakubiak and Feeney (2016) showed that secure spousal attachment predicted hedonic well-being through enhanced perceptions of goal progress. This finding aligns with cybernetic models of behavior (Carver and Scheier, 1982), which hold that positive emotions arise when individuals perceive that they are moving toward goals more quickly than expected. Extending this logic, we hypothesize that secure attachment may foster authentic-durable happiness by enhancing perceptions of PM, which provides a richer experiential account of exploration than goal progress alone.

### Hypothesis 2 (model 2: the eudaimonic/self-determination theory perspective)

From a eudaimonic perspective, happiness is conceived not merely as transient positive affect but as optimal functioning based on the satisfaction of basic psychological needs. Basic Psychological Needs Theory (BPNT) (Ryan and Deci, 2017), as part of Self-Determination Theory (SDT) (Deci and Ryan, 2008; Deci and Ryan, 2012; Ryan, 2025), posits that fulfillment of autonomy, competence, and relatedness fosters eudaimonic well-being, which in turn energizes motivation and sustained engagement. Empirical work supports this view: for example, children with higher eudaimonic well-being demonstrate greater persistence in academic tasks (Amholt et al., 2020). Because attachment theory similarly emphasizes the role of felt safety in supporting autonomy, competence, and relatedness (Jakubiak and Feeney, 2016, 2017), the two frameworks conceptually converge. Accordingly, we hypothesize that secure attachment may foster PM by enhancing authentic-durable happiness, which serves as a psychological resource that sustains engagement over time.

Attachment theory and SDT overlap conceptually in the sense that they both propose that need satisfaction is linked to happiness and achievement. More specifically, attachment theory’s basic safety need echoes: (a) SDT’s need for relatedness, as the safety need involves the need to rely on others when needed and, thus, to develop strong connections with others; (b) SDT’s need for autonomy, as the safety need involves the need to be validated in one’s choices and in one’s self-selected goals; and (c) SDT’s need for competence, as the safety need involves the need to be supported while pursuing desired goals (Jakubiak and Feeney, 2016, 2017). Moreover, attachment theory’s notion of autonomous engagement with the environment overlaps with SDT’s concept of autonomous motivation, defined as “…a motivational state in which self-initiation and coordination of personally endorsed behaviors predominate” (Weinstein et al., 2011, p. 527).

## The present study

The primary goal of this study was to examine how secure attachment relates to both PM and authentic-durable happiness, and to test two competing explanations of their interrelations. To this end, we developed and compared two structural equation models. Model 1, derived from the hedonic perspective, tested whether PM mediates the positive association between secure attachment and authentic-durable happiness. Model 2, grounded in the eudaimonic and SDT perspectives, tested whether authentic-durable happiness mediates the link between secure attachment and PM.

Our work makes two contributions. First, it extends attachment theory by positioning PM as a phenomenological manifestation of exploration that captures more than discrete perceptions of goal progress. Second, it advances well-being research by distinguishing between hedonic and authentic-durable happiness, and by examining whether secure attachment fosters enduring forms of happiness that, in turn, sustain PM.

Given the cross-sectional design and retrospective reporting period, we interpret our findings as preliminary and exploratory. Structural equation modeling is employed not to establish causality but to contrast two theoretically plausible models. Accordingly, our approach is comparative rather than confirmatory: we expect positive associations among secure attachment, PM, and authentic-durable happiness, but view model comparisons as initial evidence to guide future research.

## Method

### Transparency and openness information

We report below how we determined our sample size, all procedures, and all measures in the study, and we follow JARS. All data, analysis code, and research materials can be shared upon request. No data was missing, and no data were excluded from the analyses. Data were analyzed using XLSTAT software (version 2021.3.1.1185). This study’s design and its analysis were not pre-registered.

### Participants

The sample size for this study was calculated using G*Power software (version 3.1.9.7). A sample size of 616 was estimated based on a correlation (*ρ*) of 0.1 (small effect size), an *α* error probability of 0.05, and power (1 - *β*) of 0.80. In the present study, we were able to recruit 511 participants from the United States (381 females, 74.56%, and 130 males, 25.44%; *M*_age_ = 34.68, *SD*_age_ = 9.64, range = 18–68 years). They were invited to participate in the study from an online platform (ClickWorker). We created a digital form that was visible and accessible for the participants for 2 weeks when connecting to the platform. They reported basic demographic indicators (Ethnicity: 11.16% African American, 6.65% Asian American, 65.56% Caucasian American, 10.96% Hispanic American, and 5.68% other). Because the average index of physical exercise was above 24 (*M* = 67.71; *SD* = 42.41), the study sample was considered “active” (see “Preliminary measures” subsection).

### Study design and procedure

The study met the principles of the Declaration of Helsinki and was performed online in a survey format. Before beginning the survey, participants received instructions. They were told that the purpose of the study was to examine feelings experienced during the last 4 weeks by active physical exercisers, and for this reason they had to be regular physical exercisers to qualify for participation. To ensure that they understood what was meant by “physical exercise,” we provided them with this description: “*Physical exercise is defined as the planned, structured, and repetitive bodily movements that someone engages in during one’s free time for the purpose of improving or maintaining physical fitness and/or health* (e.g.*, walking, jogging, running, cycling, swimming, weight training, aerobics, etc*.).” They then read that their responses would remain anonymous and that they would receive financial compensation of $0.25 after completing the survey. We asked participants not to take part in the survey if they had any concerns about the proposed compensation. We also informed participants that they had the right to terminate their participation in the survey at any time without providing any justification. When participants agreed to complete the survey, they provided informed consent and were encouraged to respond to the questions that followed in a spontaneous manner. The questionnaires comprising the survey were arranged in the following order: (a) demographics and physical exercise level; (b) measures of PM perceptions; (c) secure attachment measures; and (d) measures of happiness.

### Measures

#### Preliminary measures

We used Godin and Shephard’s (1985) questionnaire to measure participants’ physical activity levels. This questionnaire assesses the average amount of time individuals spend performing physical exercise for more than 15 min during a typical week at three levels of exercise intensity: “strenuous,” “moderate,” and “mild/light.” An index of overall exercise level is then computed as follows: *Exercise amount = (9 × x strenuous exercise unit) + (5 × x moderate exercise unit) + (3 × x light exercise unit),* with *x* being the number of times an individual typically accomplishes a unit of exercise for more than 15 min per week. A score below 14 corresponds to a status of “insufficiently active” or “sedentary”; a score between 14 and 23 corresponds to a status of “moderately active”; and a score equal to or above 24 corresponds to a status of “active.”

#### Main measures

The main study measures focused on a specific time-period that occurred during the past 4 weeks. Following the work of Diener et al. (2009), we asked participants to recall their experiences from the previous 4 weeks because such a temporal window provides “…an adequate sample of feelings, rather than focusing on a short time that might not have been representative” (Diener et al., 2009, p. 254). Moreover, an examination of the past 4 weeks was used to provide “…a balance between sampling adequacy and memory accuracy” (Diener et al., 2009, p. 254).

#### Secure attachment

To assess secure attachment, we used the 7-item Secure Attachment Scale of the State Adult Attachment Measure (Gillath et al., 2009). We adapted the items to fit the temporal context of the past 4 weeks. Examples of items include: “*During the past four weeks, I felt loved*” and “*During the past four weeks, I felt secure and close to other people*” (*α* = 0.96). The items were rated along a 7-point Likert-scale ranging from 1 (“*disagree strongly*”) to 7 (“*agree strongly*”) with a neutral midpoint of 4 (“*neutral/mixed*”).

#### Psychological momentum perceptions

To assess perceptions of PM, we adapted Koestner et al.’s (2008) measure of goal progress to the exercise setting and added items to capture the broader construct of PM. First, we presented Briki and Dagot’s (2022) definition of personal goals: “*A personal goal is a project or concern that people think about, plan for, carry out, and sometimes (though not always) complete or succeed at*,” after which participants were asked to list their most important personal goal in the exercise setting (examples of goals that participants provided included “*To lose weight*,” “*To gain muscle weight*,” “*To lower blood pressure*,” “*To feel more energized*,” and “*To improve sleep quality*”). Second, participants rated their PM regarding progress toward this goal on two dimensions: *Frequency* and *intensity*. Frequency was assessed with the item: “*During the past four weeks, how often did you experience the feeling of making progress toward this goal?*” (5-point scale: 1 = “*very rarely*” to 5 = “*very often*,” with 2 = “*rarely*,” 3 = “*sometimes*,” and 4 = “*often*”). Intensity was assessed with the item: “*During the past four weeks, how generally intense did you experience your feelings of making progress toward this goal?*” (7-point scale: 1 = “*very low*” to 7 = “*very high*”).

We assessed the frequency and intensity of perceived PM because researchers highlight time- and intensity-based dimensions as central to PM experiences (Iso-Ahola and Dotson, 2016). Similarly, Diener et al. (2009) argued that when measures do not specify time or intensity, responses can be ambiguous (e.g., rarely but very intensely *vs.* frequently but mildly). By including both frequency and intensity, our bi-dimensional method reduces this ambiguity and captures two essential facets of PM (see Table 1). We did not include duration, as retrospective recall over a four-week period would likely have produced unreliable estimates of how long participants sustained PM experiences. Accordingly, our measure should be regarded as a pragmatic proxy that taps into two core aspects of PM, while recognizing that it does not capture the construct’s full multidimensionality.

**Table 1:** Reliability indexes and Spearman’s *ρ* correlations for all latent variables.

Variable	*α*	D. G. *ρ* (PCA)	1	2	3
1. Secure attachment	−	−	−		
2. PM perceptions	0.73	0.88	0.35***	−	
3. Authentic-durable happiness	0.96	0.98	0.69***	0.39***	−

***p < 0.001 for a two-tailed test. The data of all latent variables came from a confirmatory factor analysis.

#### Authentic-durable happiness

To measure authentic-durable happiness, we employed the 13-item Subjective Authentic-Durable Happiness Scale (SA-DHS; Dambrun et al., 2012) and adapted it to fit the temporal context of the study (i.e., the past 4 weeks). The SA-DHS comprises two subscales, namely “contentment” (e.g., “*During the past four weeks, what was your regular level of pleasure?*”; *α* = 0.96) and “inner peace” (e.g., “*During the past four weeks, what was your regular level of peace of mind?*”; *α* = 0.95). The items were rated along 7-point Likert scales ranging from 1 (“*very low*”) to 7 (“*very high*”). Contentment and inner peace corresponded to the two manifest variables of the latent variable “authentic-durable happiness” (see Table 1).

## Results

Before computing correlation, path, and mediation analyses, we transformed the manifest variable data into *z*-scores and computed a confirmatory factor analysis through structural equation modeling (see the subsection “Mediation and model comparisons”). In this way, we obtained the data for the subsequent latent variable analyses.

### Relationships between variables

According to Vinzi et al. (2010), a latent variable effectively reflects a set of manifest variables when the Dillon-Goldstein’s rho (D. G. *ρ*) index (determining the composite reliability of the latent variable) is greater than 0.70: all D. G. *ρ* values met this requirement (see Table 1), thus allowing us to compute correlation analyses. To examine relationships between all latent variables (secure attachment, perceptions of PM, and authentic-durable happiness), we performed Spearman’s *ρ* correlation analyses (weak effect: *ρ =* 0.10 to 0.29, moderate effect: *ρ =* 0.30 to 0.49, strong effect: *ρ =* 0.50 to 1). As predicted, the correlation analyses revealed that: (a) secure attachment was moderately related to perceptions of PM (*ρ =* 0.35, *p* < 0.001) and strongly related to authentic-durable happiness (*ρ =* 0.69, *p* < 0.001); and (b) perceptions of PM were moderately related to authentic-durable happiness (*ρ =* 0.39, *p* < 0.001) (see Table 1).

### Mediation and model comparisons

We computed structural equation model analyses (XLSTAT 2021.3.1.1185; Partial Least Squares Path Modeling [PLSPM] method: bootstrap resamples = 1,000, blindfolding = 30, and confidence intervals = 95%) to assess the fit of the built models and to compute path and mediation analyses. The PLSPM method, which refers to a non-parametric statistical technique that aims to explore (rather than confirm) a set of linear relationships between latent variables (Vinzi et al., 2010), comprises two models: the *measurement model*, which describes the links between the manifest variables and their respective latent variables, and the *structural model*, which describes the links between the latent variables (e.g., Crocetta et al., 2021). We employed the PLSPM method because of the exploratory nature of the present study.

First, the PLSPM was assessed through R^2^ and several goodness of fit (GoF) indices, such as absolute GoF (overall quality of the measurement and structural model), relative GoF (transformation of the absolute GoF), outer model GoF (measurement model quality), and inner model GoF (structural model quality) (Chin, 1998; Henseler, 2018). Values of GoF index range between 0 (model rejection) and 1 (model validation), and they must be equal to or above 0.90 for relative GoF, outer model GoF, and inner model GoF to be considered acceptable (Vinzi et al., 2010). Regarding absolute GoF, a value equal to or above 0.01, 0.25, and 0.36, respectively, refer to a small, moderate, and high overall quality model fit (Antioco et al., 2008).

Second, the PLSPM provided standardized path coefficients (*β*s) that were estimated *via* OLS regressions. A standardized path coefficient indicates the strength of a causal link that can be interpreted through the *f*^2^ coefficient (weak effect: *f*^2^ *=* 0.02 to 0.14, moderate effect: *f*^2^ *=* 0.15 to 0.34, strong effect: *f*^2^ *>* 0.35). To conclude in favor of the existence of mediation, several conditions must be satisfied: (a) the direct link between the IV and the DV (without the mediator) must be statistically significant; (b) the mediator must be significantly linked to the IV and DV; and (c) the indirect and total effects (including the mediator) must be significant. Moreover, to suggest the presence of partial or full mediation the mediation test has to produce a score of Variance Accounted For (VAF = indirect effect / total effect × 100) either above 20% (partial) or above 80% (full) (Hair et al., 2017).

**Figure 1:**
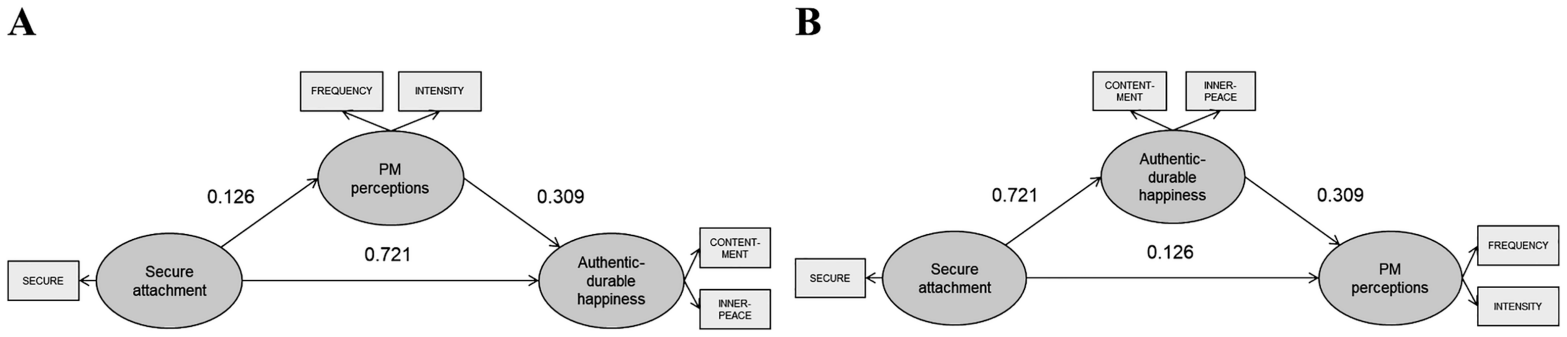
Structural equation models. **(A)** Model 1 and **(B)** Model 2. All coefficients are standardized, and solid lines indicate statistical significance.

*Hypothesis 1 (Model 1)*. The test of Hypothesis 1 indicated that Model 1’s strength was moderate, R^2^ = 33.29% (being between 33% and 67%), with acceptable goodness of fit scores (absolute GoF = 0.54; relative GoF = 0.98; outer model GoF = 1; inner model GoF = 0.98) (see Figure 1A). All mediation conditions were met; however, the VAF score was 8.16%, well below the commonly recommended 20% threshold for meaningful mediation (Hair et al., 2017). Thus, although the indirect path reached statistical significance, the effect size was too small to be substantively meaningful. In sum, the analyses did not support the mediating role of PM perceptions (see Table 2).

**Table 2:** Path estimates of model 1.

Effects	Path	*β*	SE	*t*-values	*p*-values	*f* ^2^
Direct (without mediator)	Secure attachment → Authentic-durable happiness	0.72	0.03	23.42	0.000	1.08
Mediating	Secure attachment → PM perceptions	0.35	0.04	8.38	0.000	0.14
	PM perceptions → Authentic-durable happiness	0.17	0.03	5.28	0.000	0.05
	Secure attachment → Authentic-durable happiness (direct effect)	0.66	0.03	20.72	0.000	0.85
	Secure attachment → Authentic-durable happiness (indirect effect)	0.06	0.01	4.32	0.000	-
	Secure attachment → Authentic-durable happiness (total effect)	0.72	0.02	30.36	0.000	-

Variance accounted for = 8.16%.

*Hypothesis 2 (Model 2)*. The test of Hypothesis 2 indicated that Model 2’s strength was moderate, R^2^ = 34.32%, with acceptable goodness of fit scores (absolute GoF = 0.55; relative GoF = 0.95; outer model GoF = 1; inner model GoF = 0.95) (see Figure 1B). All mediation conditions were satisfied and, notably, the VAF score was 63.83%, thereby providing evidence for the partially mediating role of authentic-durable happiness in accounting for the positive relationship between secure attachment and perceptions of PM (see Table 3).

**Table 3:** Path estimates of model 2.

Effects	Path	*β*	SE	*t*-values	*p*-values	*f* ^2^
Direct (without mediator)	Secure attachment → PM perceptions	0.35	0.04	8.39	0.000	0.14
Mediating	Secure attachment → Authentic-durable happiness	0.72	0.03	23.45	0.000	1.08
	Authentic-durable happiness → PM perceptions	0.31	0.06	5.28	0.000	0.05
	Secure attachment → PM perceptions (direct effect)	0.13	0.06	2.16	0.031	0.009
	Secure attachment → PM perceptions (indirect effect)	0.22	0.05	4.80	0.000	-
	Secure attachment → PM perceptions (total effect)	0.35	0.04	8.50	0.000	-

Variance accounted for = 63.83%.

## Discussion

In this study, we explored two models: (a) the potential link between secure attachment and perceptions of PM that, in turn, promotes authentic-durable happiness (Model 1); and (b) the potential link between secure attachment and authentic-durable happiness that, in turn, bolsters perceptions of PM (Model 2). Our expectations of positive associations among secure attachment, authentic-durable happiness, and PM were corroborated by correlation analyses, revealing moderate to strong relationships among these variables (see Table 1). Regression analyses further revealed a strong link of secure attachment with authentic-durable happiness, and exhibited a weak link with perceptions of PM (see Figure 1). Additionally, authentic-durable happiness (or perceptions of PM) weakly predicted perceptions of PM (or authentic-durable happiness) (see Figure 1). These findings parallel those observed by Jakubiak and Feeney (2016), highlighting the role of spousal secure attachment in shaping both goal-directed engagement and well-being outcomes.

### Interconnections between secure attachment, happiness, and psychological momentum

Our structural equation analyses offer useful insights into the complex relationships that shape human experience. In Model 1, the study’s outcome deviated from the findings of Jakubiak and Feeney (2016), where perceived goal progress partially mediated the association between secure attachment and hedonic well-being. In our study, although the indirect path in Model 1 reached statistical significance, the effect size was negligible (VAF = 8.16%), falling below conventional thresholds for meaningful mediation (Hair et al., 2017). Thus, Model 1 did not substantively support mediation. This difference highlights the multifaceted nature of these constructs and suggests that PM may not play the same role for authentic-durable happiness as goal progress did for hedonic well-being.

Model 2 provided tentative evidence of partial mediation, with authentic-durable happiness emerging as a potential mediator between secure attachment and perceptions of PM. Given the small increase in explained variance (from 33.29% to 34.32%), these results should be interpreted with caution. Nevertheless, the finding is conceptually consistent with the idea that authentic-durable happiness, by reflecting the fulfillment of psychological needs, may serve as a resource that sustains engagement in meaningful goals. This interpretation resonates with recent theorizing that conceptualizes PM as an adaptive, integrative process that coordinates affect, cognition, and behavior over time (Briki and Markman, 2018; Honey et al., 2023). It also suggests that while hedonic well-being, as studied by Jakubiak and Feeney (2016), may stem from self-regulatory mechanisms in goal pursuit, authentic-durable happiness may be more closely linked to the fulfillment of psychological needs (e.g., Deci and Ryan, 2008; Deci and Ryan, 2012; Ryan and Deci, 2017).

Taken together, the results from both models underscore the beneficial and enduring role of secure attachment in shaping adaptive developmental pathways. This interpretation is supported by longitudinal evidence showing that secure attachment continues to predict hedonic well-being into adulthood (Blake et al., 2024). Complementary cross-sectional research further indicates that secure attachment may foster both hedonic and eudaimonic well-being in adolescents and emerging adults (Valarezo-Bravo et al., 2024).

### Spectrum of happiness: fluctuating to authentic-durable happiness

The concept of well-being encompasses a spectrum that ranges from hedonic to eudaimonic orientations, each offering distinctive insights into the complexities of human experiences. Hedonic well-being—characterized by the evaluation of temporary positive and negative emotions alongside life satisfaction (Chui and Wong, 2016; Diener et al., 2017)—shares similarities with the concept of *fluctuating happiness* proposed by Dambrun and colleagues. This concept is driven by the pursuit of pleasure and avoidance of displeasure, resulting in emotional shifts contingent upon external factors (Dambrun et al., 2012; Dambrun and Ricard, 2012). Conversely, the concept of eudaimonic well-being—which emphasizes a life of purpose, meaning, and personal growth—aligns harmoniously with authentic-durable happiness. This form of happiness represents a sustained and stable state of contentment grounded in tranquility and peace of mind.

Aligned with attachment theory and SDT, Model 2 suggests that the fulfillment of basic psychological needs, particularly the need for safety, nurtures authentic-durable happiness, subsequently triggering motivational states toward meaningful goals. Attachment theory asserts that emotional bonds rooted in safety foster emotional comfort and self-assurance. These principles are similarly applicable to authentic-durable happiness, promoting active engagement with the surroundings (as reflected by PM) (Bowlby, 1982). SDT, functioning as a metatheoretical framework, further amplifies this perspective by asserting that the satisfaction of autonomy, competence, and relatedness needs yields positive outcomes such as eudaimonic well-being and motivation (Ryan, 2025; Ryan and Deci, 2017). This suggests that authentic-durable happiness, nurtured by the fulfillment of psychological needs, could act as a catalyst for autonomous motivation directed toward goal achievement. This synergy is extended by BPNT, which suggests that directly fulfilling needs inherently nurtures eudaimonic well-being, thereby fostering autonomous motivation for the pursuit and attainment of goals (Deci and Ryan, 2012).

Consequently, one can suggest that secure attachment may serve as a foundation for authentic-durable happiness, as it can provide a secure base from which individuals can confidently explore their potential and pursue goals that align with their values. This highlights the enduring strength of secure attachment in sustaining well-being: its benefits extend beyond everyday contexts of growth and goal pursuit to challenging circumstances (Blake et al., 2024), where secure attachment buffers against declines in mental health (Yang et al., 2024; Walsh et al., 2019). The convergence of these theoretical threads underscores the resonance between the findings of our study and those of Jakubiak and Feeney (2016). While the latter investigated fluctuating happiness, our study sheds light on the domain of enduring and authentic happiness. This complementary exploration offers a more comprehensive portrayal of the intricate fabric of well-being, encompassing both transient emotional shifts and stable contentment, and how these dynamics may interact with the pursuit of meaningful goals. The framework of positive psychology emphasizes the promotion of well-being beyond the mere absence of distress, emphasizing strengths, virtues, and the cultivation of a fulfilling life. Applying this lens provides additional insights into the alignment between eudaimonic well-being and authentic-durable happiness, offering a comprehensive understanding of how positive psychological states intertwine with PM.

## Conclusion

To conclude, the present study delved into the aspect of PM through the lens of attachment theory, suggesting not only the role of happiness in facilitating goal attainment but also emphasizing the connection between PM and exploratory behavior. Furthermore, this study provided empirical support for BPNT’s proposition that the fulfillment of psychological needs fosters the development of eudaimonic well-being (Ryan and Deci, 2017), aligning with prior research that demonstrated the promotion of autonomous engagement with activities through eudaimonic well-being (Amholt et al., 2020; Deci and Ryan, 2012). Like these preceding studies, our findings contribute to enhancing the richness of BPNT by elucidating a psychological trajectory from need satisfaction to well-being to motivation for task completion to the eventual achievement of goals.

### Limitations and theoretical perspectives

Notwithstanding, this study has limitations, and chief among them is its cross-sectional nature. Consisting of only a one-time measurement, this design hardly permits us to establish cause-and-effect links between the variables under study and does not allow us to reveal psychological trends over a period of time. Thus, further investigations should employ longitudinal designs to provide further causal evidence for the relationships examined in this study. Another limitation is the shortfall relative to the *a priori* power estimate. While G*Power calculations indicated that a minimum of 616 participants would be needed to detect a small effect size with sufficient power (*ρ* = 0.10, 1 – *β* = 0.80), the final sample included only 511 participants. To assess adequacy, we conducted a sensitivity analysis with the achieved sample size. Results showed that the study retained sufficient power (1 – *β* > 0.80) to detect correlations of approximately *ρ* ≥ 0.13 (small-to-moderate range). Thus, while the shortfall may have reduced the precision of the analyses and increased the risk of Type II error, the sample size was adequate for detecting effects in the small-to-moderate range. In this context, any findings approaching significance should be interpreted with caution; nevertheless, all relationships investigated in our study reached statistical significance.

Another limitation concerns the measurement of PM. Our approach relied on two items (frequency and intensity), chosen because they are considered central facets of PM and could be assessed with clarity. Nevertheless, this operationalization remains partial. Earlier work (e.g., Vallerand et al., 1988) attempted to capture PM through a broader multidimensional scale including notions such as synchronism, discouragement, control, confidence, motivation, energy, etc. However, several items are conceptually ambiguous, and the instrument has not been validated in exercise contexts. More recent studies have operationalized PM primarily through perceived goal progress, yet this risks conflating PM with the narrower construct of goal progress itself. Our two-item proxy therefore reduces some of this conceptual overlap but cannot capture the full phenomenological depth of PM. Future research should build and validate multidimensional PM scales that are both theoretically coherent and empirically distinct from goal progress.

In terms of theoretical development, the study provides promising avenues for future research. Because both Model 1 and Model 2 evidenced positive relationships between secure attachment, happiness and PM, future studies could follow the perspectives developed within either Model 1 or Model 2. In line with Model 1, further work could examine the effect of contexts satisfying the need for safety on fluctuating happiness through the experience of PM over several weeks. By contrast, and in line with Model 2, further work could examine whether supportive contexts developing safety feelings foster PM *via* authentic-durable happiness over time. This perspective could be tested through interventions or longitudinal designs examining the role of psychological safety and lasting well-being.

Another promising research avenue could be to examine the relationships between authentic-durable happiness, PM, and fluctuating happiness. Drouin et al. (2019) recently found that even though eudaimonic well-being and hedonic well-being can influence each other, eudaimonic well-being predominantly influences hedonic well-being, thereby supporting the predominance of the link between authentic-durable happiness and fluctuating happiness. This finding is also consistent with our view that authentic-durable happiness should be especially generated by need satisfaction, whereas fluctuating happiness should be especially generated by goal-directed perceptions (e.g., PM, self-efficacy). Following this perspective, future investigations could examine the notion that authentic-durable happiness would precipitate motivation for task completion and, thus, PM perceptions, which in turn would promote fluctuating happiness.

Furthermore, while the present study focused on psychological processes, a promising future step might be to investigate their potential neurobiological underpinnings. Secure attachment may not only promote feelings of safety and autonomy but could also shape brain systems involved in emotional regulation, resilience, and sustained motivation. Emerging neuroscience suggests that attachment experiences influence neural plasticity and stress regulation (Schore, 2001, 2009; Teicher and Samson, 2016; Tottenham and Galván, 2016), which may in turn support the conditions necessary for authentic-durable happiness and the maintenance of PM. Although no neurobiological measures were collected here, framing PM within an integrative biopsychosocial perspective opens novel avenues for interdisciplinary research. This perspective highlights the possibility that enduring happiness and motivational engagement are not only subjective experiences but may also be scaffolded by adaptive brain processes (Ryan, 2025)—an idea that, if supported by future empirical work, could significantly deepen our understanding of how attachment shapes lifelong flourishing.

### Practical perspectives

From an applied perspective, the findings of this study offer potential strategies to enhance individuals’ perceptions of PM and overall happiness. Nurturing secure attachment relationships can establish an environment conducive to emotional safety, self-assurance, and confidence (Mikulincer and Shaver, 2007). Developing supportive networks that reinforce these aspects may propel individuals toward successful goal attainment and heightened happiness (Seligman, 2002). By emphasizing the cultivation of strong emotional bonds and encouraging a supportive social environment, fitness instructors and managers could facilitate an atmosphere that promotes heightened psychological well-being and boosts motivation for personal goal pursuit (Ryan and Deci, 2000; Ryan, 2025).

The distinction between authentic-durable happiness, characterized by sustained contentment, and fluctuating happiness driven by fleeting emotional responses, underscores the importance of prioritizing practices that nurture lasting well-being (Hefferon and Boniwell, 2011). Encouraging activities aligned with psychological needs, mindfulness, and introspection can cultivate inner peace and emotional stability (Sheldon and Lyubomirsky, 2006). Integrating these practices into interventions holds promise for instilling a lasting foundation from which individuals can approach goal pursuit with sustained enthusiasm. The synthesis of attachment theory and SDT provide insights that can guide intervention design (Reis and Patrick, 1996). Recognizing the power of fulfilling fundamental psychological needs—autonomy, competence, and relatedness—can act as a catalyst for authentic-durable happiness and intrinsic motivation (Deci and Ryan, 2000; Ryan, 2025). By cultivating environments that foster these core needs, instructors can invigorate exercisers’ well-being and propel them towards goal attainment, driven by their sense of capability, connection, and self-directedness (Deci and Ryan, 2000; Ryan, 2025).

Considering the bidirectional interplay between transient and enduring happiness, tailoring exercise settings to uplift momentary, or hedonic, well-being emerges as an aligned approach for focused interventions. Creating environments that amplify positive emotions and provide incremental steps toward achieving exercise goals has the potential to significantly enhance hedonic well-being (Fredrickson, 2001). In turn, this contributes to an increased sense of overall psychological well-being. Additionally, fostering a cultural environment that acknowledges and celebrates even small accomplishments can actively nurture positive emotional experiences, reinforcing the enduring experience of genuine happiness (Lyubomirsky, 2008).

In conclusion, this study reveals the interplay between attachment, well-being, and goal attainment, highlighting the role of psychological factors (Bowlby, 1982). By aligning these factors, instructors or educators evolve into more than just promoters of well-being; they become guides, assisting exercisers in moving toward achievement and profound happiness. This study offers a pathway toward realizing aspirations, bolstered by enduring well-being and robust emotional resilience.

## Data Availability

The raw data supporting the conclusions of this article will be made available by the authors, without undue reservation.
